# Supplementary material for: Effectiveness and feasibility of structured emotionally focused family therapy for parents and adolescents: Protocol of a within-subjects pilot study
Source: PLoS One. 2023 Jun 23;18(6):e0287472. doi: 10.1371/journal.pone.0287472 (PMC10289383; doi:10.1371/journal.pone.0287472)
Supplement: S1 File — (DOCX) [file pone.0287472.s001.docx]

**Roadmap structured EFFT**

| **Phase 1 Family: relational reframe** | |
| --- | --- |
| **Goal 1**: Developing the working alliance. | |
| **Goal 2**: Assessment of destructive interaction patterns, awareness escalation. | |
| **Goal 3**: Relational reframe of problems at intake, consensus on relationship recovery as treatment goal. | |
|  |  |
| **Phase 2A Parents: deepening of emotions and preparation** | **Phase 2B Child: deepening of emotions and preparation** |
| If necessary repeat goals 1, 2 and 3  **Goal 4**: Experiencing vulnerable emotions underneath hyperactivation and deactivation. | If necessary repeat goals 1, 2 and 3  **Goal 4**: Experiencing vulnerable emotions underneath hyperactivation and deactivation (plus understanding of problems at intake in the context of attachment history). |
| **Goal 5**: Increasing availability and responsiveness starting from vulnerable emotions. Three relationship levels: | **Goal 5:** Exploring needs for validation and support starting from these vulnerable emotions. |
| (a) relationship parent-own parents. Goal to understand own reactivity from attachment history, sub goals: |  |
| (a1) understanding and reduction of own reactivity; |  |
| (a2) develop an understanding of the child's behavior, pain and needs; |  |
| (a3) reduction of self-blame. |  |
| (b) relationship partner-partner. Goal is to make attachment more secure, because: |  |
| (b1) models emotional regulation in children; |  |
| (b2) is a base for secure attachment between parent-child; |  |
| (b3) is the base for a stronger parental alliance. |  |
| (c) relationship parent-child. Goal is to make attachment more secure (available and responsive). |  |
| **Goal 6:** Prepare attachment repair conversations and enactments with the child. | **Goal 6:** Prepare attachment repair conversations and enactments with the parents. |
|  |  |
| **Phase 3 Parents and child: repair of secure attachment in enactments** | |
| **Goal 7:** Repair of secure attachment between parents and child in enactments. | |
|  |  |
| **Phase 4 Family: consolidation** | |
| **Goal 8:** Consolidation of new interaction patterns within family. Involve brothers and sisters.  **Goal 9:** Use safe interaction patterns as base to deal with residual problems and symptoms. | |
