# Supplementary material for: Effectiveness and feasibility of structured emotionally focused family therapy for parents and adolescents: Protocol of a within-subjects pilot study
Source: PLoS One. 2023 Jun 23;18(6):e0287472. doi: 10.1371/journal.pone.0287472 (PMC10289383; doi:10.1371/journal.pone.0287472)
Supplement: S1 Protocol — (PDF) [file pone.0287472.s003.pdf]

# Effectiveness and feasibility of structured Emotionally Focused Family Therapy: A pilot study

## Project details

**ERB number** 2022-CP-15102
**ID** 15102
**Title** Effectiveness and feasibility of structured Emotionally Focused Family Therapy: A pilot study
**Department** Clinical Psychology
**Status** Approved
**Created** 2022-04-22 14:03:30
**Modified** 2022-04-24 07:04:22

## Project collaborators

Henk Jan Conradi Owner h.j.conradi@uva.nl 0205258606
Arnold van Emmerik Ethics Member a.a.p.vanemmerik@uva.nl 0205258604

## General

**Responsible researcher**

Henk Jan Conradi

**Who conducts the research?**

Henk Jan Conradi is the principal investigator. Therapists on location supervised by L. Rodenburg (Psychologiepraktijk Lenny Rodenburg) administer and collect questionnaires. Interviews will be conducted by Trudy Mooren (Universiteit Utrecht) and Daphne Meuwese (SGGZ Altrecht). Master students will assist.

**Research location**

Five private practices under supervision of Lenny Rodenburg (Psychologiepraktijk Lenny Rodenburg).

**Research category**

Research staff

**Brief project description**

With this pilot study we want to obtain preliminary insight in the feasibility and effectiveness of structured Emotionally Focused Family Therapy (EFFT). EFFT aims at the development of secure attachment between parents and their children in order to reduce the child's vulnerability for mental health problems. In this way EFFT facilitates the creation of a base of resilience for children characterized by functional emotion regulation and enhanced self-esteem and trust in others on which they can thrive (Furrow et al., 2019). The design will be a non-randomized within subjects design. Effectiveness will be tested by comparing change on self-report questionnaires during the two month waiting-period with change during the four months treatment phase that follows the waiting period. Maintenance of treatment change will be tested by comparing post-treatment scores with follow-up self-report scores two months after closure of treatment. Feasibility will be assessed by means of a semi-structured interview after follow-up. Treatment adherence will be evaluated by scoring of audio recordings of random treatment sessions with observation checklists based on the treatment protocol (Rodenburg, Meuwese & Conradi, 2022) .

**Expected duration of the project**

Approximately three years.

**Expected number of participants**

15

We aim at 15 to 20 included families.

**Lab Guide and Data Storage Protocol**

Yes

**Expected participation duration**

90

5 sessions with questionnaires of each approximately 60 items (5 * 12 minutes), plus an interview of approximately 30 minutes.

**Reward Type**

None (please explain in a comment)

Families will invest 90 minutes over 7 months. With this they facilitate the development of structured EFFT.

**Online research**

Yes

The pre-waiting period questionnaire will be administered online with Qualtrics. The other 4 questionnaires will be administered by paper and pencil in the therapy room

of the therapist. Additionally, a semi-structured online interview concerning the feasibility of structured EFFT will be administered at the end of follow-up.

**This project is comparable with the following submitted project(number)**

## Privacy

**Will you collect or process data from human participants?**

Yes

**What personal data relating to subjects are recorded?**

E-mail
Age / Date of birth
Gender
Education
Facial images/video/sound recordings

No facial images or video recordings, audio recordings only.

**What subject groups are relevant?**

Subjects with age between 12 and 16
Other, please explain in a comment below

Adolescents between 12 and 16, and 16 and 18, and adults (the parents).

**Are the subjects in the study actively consenting to participation?**

Yes

**Responsible party**

Myself, as a researcher employed by the UvA or myself in collaboration with UvA colleague(s)

**Who will be processing the data?**

Myself, as a researcher employed by the UvA together with an external non-commercial party in the EU
(e.g., researchers from another EU-university (please supply contact information in a comment below))

Myself in collaboration with Trudy Mooren of the University of Utrecht and Daphne Meuwese (SGGZ Altrecht).

**Legal agreement data processing?**

Yes

Seven agreements are produced by the legal department of the UvA in cooperation with the data steward of Psychology: one with the University Utrecht and one with SGGZ Altrecht, and five agreements with the five private practices we work together with.

**Where are the data to be stored?**

Other, please explain in a comment below

Research drive. Scores on pen and paper self-reports will be double checked and stored in SPSS files on Researchdrive.

**What security measures have been taken?**

Pseudonymisation (identifying data has been replaced with pseudonyms)
Other, please explain in a comment below

I contacted several data officers and stewards, i.e, Tinka Beemsterboer, Marco Teunisse and Miek Krol. Together we completed the DPIA questionnaire. All data, self-report questionnaires, semi-structured interviews and sound recordings will be stored on Researchdrive which is the most secure storage option at the moment. Concerning the three data sources, the following procedures are agreed upon with

the data stewards: (1) Self-report questionnaires. (a) Pre-waiting self-report questionnaires will be completed in Qualtrics. Final data files will be stored on Researchdrive. Subsequently, the Qualtrics files will be deleted. (b) Pre-, mid- and post-treatment and pre-booster are paper and pencil questionnaires. score will be entered in pseudonimized SPSS files by master students and uploaded to Researchdrive. Subsequently, the paper forms will be destroyed (blue sealed containers) or stored by UvA DIV (via TOP, Rene van der Belt). (2) Sound recordings of random sessions. Therapists will make sound recordings with an encrypted dictaphone. They will not use family names etc. but only an ID number to identify the family. After recording they will upload the recording to Researchdrive. Subsequently, they will delete the original recording on the dictaphone. Therapists cannot download files. Students will score the sound recordings while playing them from Researchdrive. Students are not able nor allowed to download them. (3) Semistructured interviews after the booster session. Trudy Mooren (UU) and Daphne Meuwese (SGGZ Altrecht) will conduct these interviews online via Zoom. Zoom is the safest as their meetings are end to end encrypted. Sound recordings are downloaded to the laptop of one of the two researchers (Trudy Mooren or Daphne Meuwese). They will upload the interviews to Researchdrive and subsequently delete the recordings on their computers. They will score the sound recordings while playing them from Researchdrive. They are not able nor allowed to download them. Master students who assist in the project will have access to the SPSS files and sound recordings, but will not be able to download the sound recordings. They have to sign a statement in which they promise to treat all data as confidential and not to share them with anybody (social media, friends colleagues, other students etc.). The data stewards approved these procedures.

## Ethics Checklist

**A1. METC vs Local ERB (FMG-UvA)**

The WMO-act does not apply; the research can be submitted to the local EC.

**A2. Participants**

Yes, Category 2: Participants are recruited via a newspaper advert, posters or institutions

**A3. Freedom to participate**

Yes

**A4. Screening**

I hesitate and explain in a comment why

For this pilot study we will include families with (a) adolescents, i.e., between 12 and 18 years of age, as the ‘identified’ patient, and (b) families coping with mild problems. The latter means we will exclude (a) blended families, because of the more complex loyalties that exist between children and step parents; (b) families of which individual members cope with serious trauma such as sexual and physical abuse and severe neglect; and (c) families of which the parents or children are diagnosed with severe DSM disorders (substance abuse or psychosis). Exclusion criteria will be determined with unstructured clinical interviews by experienced clinicians during the screening.

**A5. Chance Incidents**

No, the method precludes chance incidents

**A6. Information and informed consent**

Yes, please submit the information letter and the consent form as attachment

**A7. Anonymity and privacy**

Yes

**A8. Deception and debriefing**

There is no deception

**A9. Discomforting research**

No

The research itself consists of self-report questionnaires, an interview concerning feasibility of EFFT and sound recordings of random EFFT sessions, which are in itself not discomforting.

**B1 Does the research fully comply with the guidelines for Standard Research?**

B2. Standaardonderzoek programmagroep Ontwikkelingspsychologie

## Pre-registration

**Have you already pre-registered**

No

**Is the study exploratory, confirmatory, or a combination?**

combination of exploratory and confirmatory

**Hypothesi(e)s**

Concerning the effectiveness of structured EFFT we hypothesize that outcomes will show: (1) no, or less change during the waiting period compared with the treatment phase; (2) gain during the treatment phase; (3) and substantial maintenance during follow-up. Feasibility will be explored.

**Data collection procedures**

For this pilot study we will include families with (a) adolescents, i.e., between 12 and 18 years of age, as the ‘identified’ patient, and (b) families coping with mild problems. The latter means we will exclude (a) blended

families, because of the more complex loyalties that exist between children and step parents; (b) families of which individual members cope with serious trauma such as sexual and physical abuse and severe neglect; and (c) families of which the parents or children are diagnosed with severe DSM disorders (substance abuse or psychosis). Exclusion criteria will be determined with unstructured clinical interviews by experienced clinicians during the screening. Families are recruited by five private EFT practices.

**Sample size**

We aim at 15 to 20 families. At level 3 we will analyze three types of dyads (i.e. mother-adolescent, father-adolescent and the parents as partners), level 2 consists of the individual respondents (adolescent, mother and father) and level 1 of the repeated measurements (five time points). The study is designed as a pilot study and will therefore be underpowered.

**Research methodology and design**

A within-subjects design with three waves without randomized control group will be used. The waiting period will span approximately two months, the treatment phase will last three to four months and will consist of 16-21 protocolized sessions, followed by a two months follow-up period concluded with a booster session. A randomized controlled design was deemed not feasible for this pilot study, we therefore opted for a within-subjects design in which families will serve as their own controls. By comparing change during the waiting period with change during the treatment phase we will get a clear impression of spontaneous remission vs. treatment-related change. The study will use a multi-method approach. For the effectiveness part quantitative methods (quantitative analyses concerning self-report questionnaires) will be applied and for the feasibility part qualitative methods (qualitative analyses of semi-structured interviews). Dependent variables will be: Negative interaction patterns within the three dyads will be measured by the Relationship Dynamics Scale (RDS; Stanley et al., 2001) a four item self-report questionnaire. Father and mother will complete the RDS twice per assessment (for their relationship with their partner and for their relationship with their child). The adolescent will complete the RDS twice as well (for their relationship with their mother and their father). Accessibility and Responsiveness of the attachment figures assessed with a brief version consisting of six items of the Accessibility, Responsiveness, Emotional Engagement questionnaire (ARE; Johnson, 2008). The adolescent will complete the ARE two times per assessment (separately for the relationship with the mother and the father). Emotional Availability and Discipline as assessed with the Self-Efficacy for Parenting Tasks Index Toddler Scale (SEPTI-TS; Coleman & Karraker, 2003). The parents will complete this questionnaire for their relationship with their child. Attachment will be assessed with the Experiences in Close Relationships - Relationship Structures questionnaire (ECR-RS; Fraley et al., 2011). The adolescent will answer the questions twice, separately for the mother and the father. The parents complete the questionnaire only for their relationship with their partner, but not for their relationship with their adolescent child as the child is no attachment figure for the parents. Relationship Satisfaction between the parents as partners will be measured with the four items of the Couple Satisfaction Index (CSI-4; Funk & Rogge, 2007). Adolescent’s complaints will be assessed with the Strengths and Difficulties Questionnaire (SDQ; Goodman, 1997). We will administer the version for children aged 4-17 to be completed by the adolescents, and the parents version for the parents. We will use the total score which is labelled ‘Total difficulties’ and made up of the scores on all subscales minus Prosocial behavior and the subscales Internalization and Externalization. These dependent variables

are measured at five time points: pre-waiting period, pre-treatment, prior to phase 3 of EFFT (all questionnaires except for the SDQ), post treatment and prior to the booster session. Feasibility of EFFT will be explored with a semi-structured interview with each of the parents and the adolescent conducted after the booster session. The semi-structured interview will last approximately 30 minutes per family member. Each baseline variable can be used as predictor variable. In that case the baseline score of the outcome plus baseline score * time have to be included as covariates in order to adjust for room for improvement effects. Covariates for the main analyses might be age, SDQ etc.

**Analysis**

Linear Mixed Models will be used to examine the main hypotheses.

**Other**

## Attachments

**Add a concise research description (max 1 A4) and any other relevant documents.**

1. 2022-06-08 research description EFFT pilot study.docx

**Information letter for the participant**

1. 2022-06-08 informatiebrief adolescent 16 tm 18 jaar.docx
2. 2022-06-08 informatiebrief ouders plus adolescenten 12 tot 16 jaar.docx
3. 2022-06-08 informatiebrief ouders.docx

Informed consent and information letter will be administered on Qualtrics.

**Informed Consent Form for the participant**

1. 2022-06-08 informed consent adolescent 16 tm 18.docx
2. 2022-06-08 informed consent ouders en adolescent 12 tot 16 jaar.docx
3. 2022-06-08 informed consent ouders.docx

## Project history

**2022-06-10 15:06:20** Project approved [by Arnold van Emmerik]

Dear Colleague,

I hereby acknowledge **receipt** of your project, archived as 2022-CP-15102.

Please use this file number in future correspondence.

Your project has been reviewed and is hereby approved.

Modifications of the concerning project should be submitted to the Ethics Review Board for evaluation.

Regards,

Arnold van Emmerik,

Member of the Ethics Review Board.

Addressed to project owner h.j.conradi@uva.nl

**2022-06-10 14:54:44** Ethics member Arnold van Emmerik (avemmer1) has been added. [by Arnold van Emmerik]

**2022-06-09 16:42:13** Project submitted for review. [by Henk Jan Conradi]

**2022-04-22 14:03:30** Project created [by Henk Jan Conradi]
