# Supplementary material for: Effectiveness and feasibility of structured emotionally focused family therapy for parents and adolescents: Protocol of a within-subjects pilot study
Source: PLoS One. 2023 Jun 23;18(6):e0287472. doi: 10.1371/journal.pone.0287472 (PMC10289383; doi:10.1371/journal.pone.0287472)
Supplement: Attachment — Submitted filename: Response to reviewers.docx [file pone.0287472.s005.docx]

PONE-D-22-28187R1

Effectiveness and feasibility of structured emotionally focused family therapy for parents and adolescents: Protocol of a within-subjects pilot study

PLOS ONE

Reviewers' comments:

Reviewer's Responses to Questions

**Comments to the Author**

6. Review Comments to the Author

Please use the space provided to explain your answers to the questions above and, if applicable, provide comments about issues authors must address before this protocol can be accepted for publication. You may also include additional comments for the author, including concerns about research or publication ethics.

You may also provide optional suggestions and comments to authors that they might find helpful in planning their study.


(Please upload your review as an attachment if it exceeds 20,000 characters)

Reviewer #1: I am pleased with how the authors addressed the majority of my initial comments. I apologize for not being explicit enough in a few of my initial comments and will clarify them so that the authors can comment and/or revise.

Comment 3
There are two interventions with the abbreviation EFFT. The Encyclopedia of Couple and Family Therapy presents the two in consecutive chapters. Emotionally focused family therapy by Furrow and Palmer and Emotion-focused family therapy by Sabey and Lafrance are the two distinct EFFTs. Many years of research support the latter. See e.g., <https://efftinternational.org/research>.

Since these two EFFTs are available today and the latter has been investigated for a number of years, I would propose that you include a section in the manuscript that comments on this so that the readers are informed and there is no misunderstanding as to which EFFT you intend to explore. If you do not include any of the research on that EFFT in your manuscript, you should also provide a reason why it is not relevant.

Reply 3

You are right. We were aware of Emotion-focused family therapy by Lafrance et al. (2020), but were not sure you meant this particular EFFT. We made clear in the text it should not be confused with *Emotionally*-focused family therapy originating with Furrow and Palmer (2019). The latter has the attachment relationship as a more central focus. Although important, we did not include the Emotion-focused family therapy studies you referred at as these studies concern the *brief group course* based on Emotion-focused therapy and not the regular therapy and as such is not comparable to EFFT examined in this study. We now write (lines 93-96):

Of note, *Emotionally*-focused family therapy, the intervention examined in this study, should not be confused with *Emotion*-focused family therapy.^18^ Emotionally-focused family therapy has attachment as its central focus, whereas Emotion-focused family therapy works with the general emotion coaching principles as described by Greenberg.

Comment 11
I appreciate your clarification. I want to confirm that I and future readers have correctly understood your meaning. Do you intend to exclude any children or parents who are undergoing therapy for whatever reason?

Reply 11

Yes.

Comment 20 
Under the guidelines for me as a reviewer, PLOS ONE asks, "Have the authors described where all data underlying the findings will be made available when the study is complete?" Since you have not stated WHERE it will be made available, I have to answer "no" to that question. I suggest you state where it is supposed to be publicly stored.  One suggestion is osf.io, and another is data.mendeley.com, but you are free to choose any other repositories.

Reply 20

We followed your suggestion (line 417).
